# In Memoriam: Robert Emmons Kissling (1923–2013)

**DOI:** 10.3201/eid2008.140672

**Published:** 2014-08

**Authors:** Charles H. Calisher, Frederick A. Murphy, Thomas P. Monath

**Affiliations:** Colorado State University, Fort Collins, Colorado, USA (C.H. Calisher);; University of Texas Medical Branch, Galveston, Texas, USA (F.A. Murphy);; Hookipa BioTech AG, Vienna, Austria (T.P. Monath);; PaxVax Inc., Menlo Park, California, USA (T.P. Monath)

**Keywords:** in memoriam, Robert Emmons Kissling, Centers for Disease Control and Prevention, CDC, virology

Robert Kissling (Figure), a pillar of US Center for Disease Control (CDC) infectious diseases programs through an era of great programmatic and institutional growth (1947–1973), died November 13, 2013 at his home in North Carolina. Through his years at CDC, Bob led investigations of great diversity and impact, always with the same sense of openness, integrity, composure, and respect for everyone involved.

**Figure:**
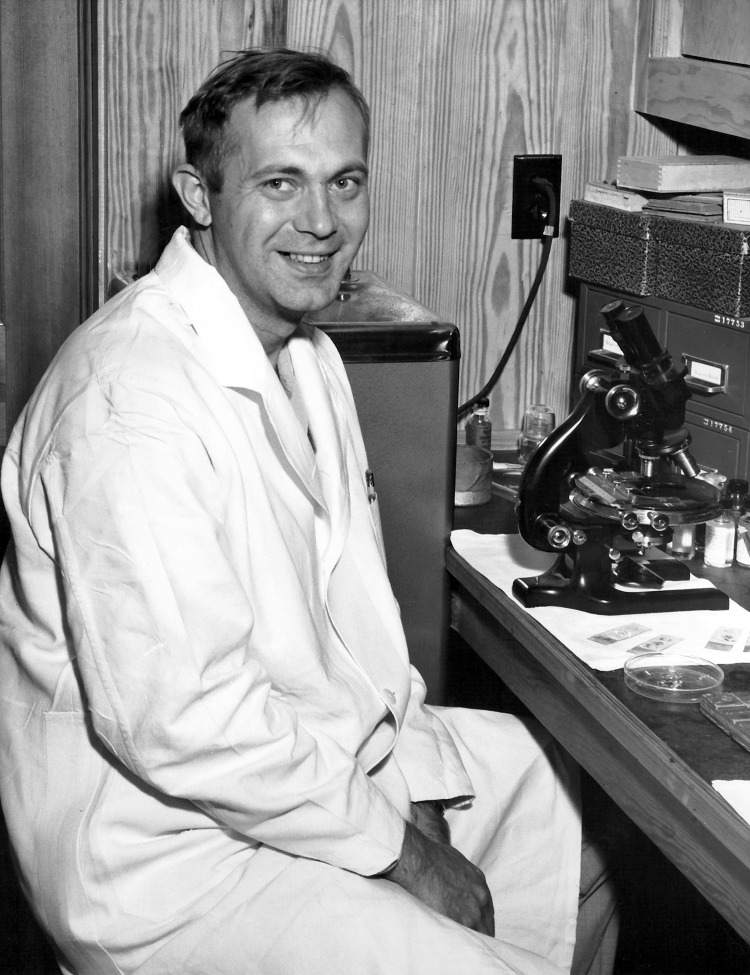
Robert Emmons Kissling

The fondest of memories return; they remind the authors, his colleagues, and friends that it was with this attitude that the viral disease programs of CDC were established and have been maintained through the years. Bob, as an eminent virologist and a leader of CDC’s viral disease programs, left a permanent legacy.

Born in Toledo, Ohio, Bob attended Otterbein College, was awarded a DVM and an MSc from the College of Veterinary Medicine, Ohio State University, and then spent a year at the graduate school of the University of California at Davis. In 1947, the year after CDC was founded, he joined the US Public Health Service and began a long and distinguished career with CDC, then known as the Communicable Disease Center.

The 1950s was a period of great progress in field and laboratory research in virology and at CDC’s Virus and Rickettsia Section in Montgomery, Alabama. Bob began collaborative studies of the natural history, epidemiology, and pathogenesis of vectorborne viral encephalitides and began a career-long interest in rabies research; CDC developed an outstanding reputation in these areas, not the least because of Bob’s contributions. While in Montgomery, Bob married Martha Eidson, his laboratory technician, and they had 3 children.

In 1958, Bob and guest researcher Robert Goldwasser succeeded in adapting the then-new immunofluorescence methodology to detect rabies viral antigen in brain specimens of animals suspected of being rabid (*1*). Field trials provided proof of its 100% sensitivity, specificity, and reliability. The assay became the first “rapid” viral diagnostic test, with results reported quickly enough to guide postexposure treatment. Remarkably, this is now the oldest rapid viral diagnostic test in use, and it remains the national and World Health Organization international standard assay. Bob’s interest in rabies continued after the Virus and Rickettsia Section was moved into CDC’s new facility in Atlanta, Georgia, in 1960. He was the first person to amplify rabies virus in modern cell culture systems; one of his cell culture–adapted virus strains was later used to produce the most popular human rabies vaccine in the United States.

In the new laboratory facilities in Atlanta, CDC’s viral and rickettsial disease programs flourished, as did Bob’s role as scientist and leader. As was the way in the 1960s and 1970s, virologists such as Bob were quite broad in their outlook and expertise. He and his colleagues worked on rabies, borreliosis, Chagas disease, psittacosis, and more, with a major emphasis on the natural history of arbovirus diseases. With his colleagues, Bob did the original field and laboratory research on eastern equine encephalitis virus, unraveling its complex transmission cycle in birds and mosquitoes.

As the years went by, Bob took on ever-increasing administrative responsibilities at CDC, serving as chief of various CDC organizational units: Veterinary Research, Viropathology, and Hepatitis Activities. From 1968 until 1973, he was chief of the Virus and Rickettsial Disease Section, Bureau of Laboratories. This period saw the emergence of several important new viral diseases (or of diseases at first thought to have a viral etiology) and the emergence of increasingly sophisticated approaches to virus research, virus detection and identification, and the means to guide CDC’s mission of disease prevention and control. Two new diseases are exemplary of the importance of his work, Marburg hemorrhagic fever and Lassa fever.

In 1967, cases of hemorrhagic fever occurred among laboratory workers in Germany and Yugoslavia who were processing kidneys for cell culture; the kidneys were from African green monkeys that had been imported from Uganda. A new virus, Marburg virus, was isolated from patients and monkeys, first in Germany but shortly thereafter at CDC. From the beginning, it was realized CDC must have the capacity to deal with this virus and that Marburg virus was dangerous, calling for the best biocontainment of the day. CDC borrowed a mobile containment laboratory from the National Institutes of Health; the containment level provided was about what would be called Biosafety Level 2+ today. The work was led by Bob (*2*); the main safety feature was that only 2 other people would be at risk, Roslyn Robinson and Fred Murphy, each experienced in safe practices for working with dangerous pathogens. The virus was obtained and used to develop diagnostic reagents and for morphologic, pathologic, and ultrastructural pathologic studies. A serologic test was developed and used to try to discern the source of the virus in Uganda. Bob uniquely foresaw the role that CDC had to play in this new field of exotic, dangerous pathogens, a field that would continue to grow in following years.

As understood clearly by Bob, the Marburg virus episode had to be the impetus for the development of permanent biocontainment facilities at CDC. A glove port cabinet line laboratory was constructed, which opened in 1968. Completion of the laboratory coincided with the emergence of Lassa fever in West Africa. Early work on Lassa virus elsewhere was terminated after 1 person died and another nearly died; all virus stocks were sent to CDC. Under Bob’s leadership, Tom Monath and others set to work to develop diagnostic systems and uncover the natural history and reservoir host of Lassa virus (*3*). Work expanded from an emphasis on diagnostics to the pathogenesis of several viral hemorrhagic fevers. All this activity put a great strain on the glove port laboratory, and soon Bob led long-gestating planning for a large higher containment laboratory. CDC’s present leadership role, globally, in dealing with dangerous pathogens stems from Bob’s initial vision.

Retiring to a farm north of Atlanta in 1973, Bob and Martha were at last able to bird-watch, farm a bit, and enjoy the wildflowers. Martha died in 1999 and Bob, whose health was declining, moved to North Carolina to enjoy his children, grandchildren, and great-grandchildren. A gentleman of common sense, practical sense, he was always polite, always helpful, always willing to teach, and greatly respected by his peers. Bob Kissling was one of the giants of CDC.
